# Reply to Brea et al. Comment on “Levitt et al. Approach to Decompensated Right Heart Failure in the Acute Setting. *J. Clin. Med.* 2024, *13*, 869”

**DOI:** 10.3390/jcm13133849

**Published:** 2024-06-29

**Authors:** Catherine V. Levitt, Caitlin A. Williams, Jalil Ahari, Ali Pourmand

**Affiliations:** 1 Department of Emergency Medicine, The George Washington University School of Medicine and Health Sciences, Washington, DC 20037, USA; catherinelevitt@gwu.edu (C.V.L.); cawilliams@gwu.edu (C.A.W.); 2 Pulmonary and Critical Care Medicine, The George Washington University School of Medicine and Health Sciences, Washington, DC 20037, USA; jahari@mfa.gwu.edu

We would like to acknowledge and thank the authors of “Defining the Plethoric IVC” [1], which comments on our original article “Approach to Decompensated Right Heart Failure in the Acute Setting” [2]. This comment adds to the conversation regarding measuring the inferior vena cava (IVC) using point-of-care ultrasound (POCUS). In our original text, we wrote that the plethoric IVC, without inspiratory collapse, is <10 mm; however, upon re-examination of our references, and the comment submitted, we feel that this may have been an error, and would like to amend it to >20 mm.

In situations where the right ventricle has been remodeled, such as in chronic pulmonary hypertension, the right ventricle has decreased compliance, which leads to elevations in right atrial pressures and chronic IVC enlargement with reduced inspiratory collapse [3]. These elevated right atrial pressures are transmitted to the IVC, which impedes the forward systolic flow, leading to reduced IVC collapsibility and, eventually, IVC dilation [3]. On POCUS, IVC dimension >21 mm, IVC respiratory non-collapsibility (<50%), and right internal jugular non-collapsibility (<50%) were associated with elevated right atrial pressures [4].

The practice of measuring the IVC using POCUS is becoming more appealing to providers in the emergency department, as it is thought to provide a quick and reliable assessment of a patient’s volume status. However, these measurements differ markedly depending on the patient’s size, positioning, manner of breathing, and measurement site, and there is a large heterogeneity of published results on this topic [5]. Similarly, there is also variability depending on which anatomical approach is utilized to obtain the IVC volumes, including subxiphoid transabdominal long axis, transabdominal short axis inferior to the hepatic veins, or right lateral transabdominal coronal long axis [5]. As the IVC is elliptical, the diameter measurements vary along the course of the vein, and though POCUS is usually performed after the confluence of the hepatic veins, it is possible the more accurate measurement is at the confluence of the renal veins, particularly in mechanically ventilated patients [6,7]. We must also take into account that there is marked variability between providers who are using POCUS to obtain IVC measurements [8]. Furthermore, disagreement exists regarding which mode to utilize to obtain IVC measurements. M-mode (motion mode) is an older ultrasound imaging modality that acquires a 2D image and measures the movement of structures over time, while B-mode (brightness mode) utilizes small ultrasound echoes to create arrays of dots to create a picture. While M-mode is popularly used to measure IVC collapsibility, there are conflicting data available to suggest whether B-mode or M-mode is superior [6,8,9].

Though POCUS is a useful tool in determining volume status and right atrial pressures in right heart failure, it is not without its limitations. While we advocate for its use in helping to aid clinicians presented with a patient in an acute right heart failure exacerbation, we reserve the right to mention that there is very little standardization when it comes to IVC measurement in this patient population. Our previously written <10 mm, is likely erroneous after further research, and the more accurate statement is an IVC diameter of >20 mm, without respiratory collapse. 
